# Involvement of both caspase-8 and Noxa-activated pathways in endoplasmic reticulum stress-induced apoptosis in triple-negative breast tumor cells

**DOI:** 10.1038/s41419-017-0164-7

**Published:** 2018-01-26

**Authors:** Ana Cano-González, Marta Mauro-Lizcano, Daniel Iglesias-Serret, Joan Gil, Abelardo López-Rivas

**Affiliations:** 1 Centro Andaluz de Biología Molecular y Medicina Regenerativa-CABIMER,, CSIC-Universidad de Sevilla-Universidad Pablo de Olavide,, Avda Américo Vespucio 24,, 41092 Sevilla,, Spain; 2 Departament de Ciencies Fisiologiques, Facultat de Medicina i Ciencies de la Salut, Universitat de Barcelona–IDIBELL (Institut d’Investigacio Biomedica de Bellvitge), L’Hospitalet de Llobregat, Barcelona, Spain; 3 Centro de Investigacion Biomédica en Red-Oncología (CIBERONC), Carlos III Health Institute, Madrid, Spain

## Abstract

Recent evidences indicate that triple-negative breast cancer (TNBC) cells with a mesenchymal phenotype show a basal activation of the unfolded protein response (UPR) that increases their sensitivity to endoplasmic reticulum (ER) stress although the underlying cell death mechanism remains largely unexplored. Here we show that both caspase-8-dependent and -independent apoptotic mechanisms are activated in TNBC cells undergoing sustained ER stress. Activation of the extrinsic apoptotic pathway by ER stress involves ATF4-dependent upregulation of tumor necrosis factor-related apoptosis-inducing ligand receptor 2 (TRAIL-R2/DR5). In addition, accumulation of BH3-only protein Noxa at the mitochondria further contributes to apoptosis following ER stress in TNBC cells. Accordingly, simultaneous abrogation of both extrinsic and intrinsic apoptotic pathways is required to inhibit ER stress-induced apoptosis in these cells. Importantly, persistent FLICE-inhibitory protein (FLIP) expression plays an adaptive role to prevent early activation of the extrinsic pathway of apoptosis upon ER stress. Overall, our data show that ER stress induces cell death through a pleiotropic mechanism in TNBC cells and suggest that targeting FLIP expression may be an effective approach to sensitize these tumor cells to ER stress-inducing agents.

## Introduction

Physiological or pathological alterations in the cellular environment can disrupt the protein folding capacity of the endoplasmic reticulum (ER), causing ER stress^1^. In vertebrates, accumulation of unfolded proteins in the ER lumen is detected by three different types of protein sensors^2–4^ located in the luminal face of the ER membrane that activate an adaptive response known as the unfolded protein response (UPR)^5^ to restore protein homeostasis in the ER. Activation of these signaling pathways leads to a reduction in the influx of proteins into the ER, activates protein degradation pathways, and increases the folding capacity of the ER^5^. However, under severe or sustained ER stress some of the UPR signaling pathways will activate a cell death process by engaging the apoptotic machinery^6,7^. Upregulation of proapoptotic proteins and downregulation of antiapoptotic proteins of the Bcl-2 family have been observed in cells undergoing apoptosis upon ER stress^8–10^. In addition, upregulation of tumor necrosis factor-related apoptosis-inducing ligand receptor 2 (TRAIL-R2/DR5) expression and activation of the extrinsic apoptotic pathway following ER stress has also been demonstrated^11–13^. However, whether or not both intrinsic and extrinsic apoptotic pathways are activated simultaneously and the relative contribution of each pathway to apoptosis in cells undergoing ER stress is an issue that remains largely unresolved.

Triple-negative breast cancer (TNBC) is a heterogeneous disease representing 10–20% of cases of breast tumors, characterized by the absence of estrogen receptors (ER−) and progesterone receptors (PR−) and lack of human epidermal growth factor type 2 receptor gene amplification^14^. TNBC has poor prognosis and a high rate of early relapse and still pose a major challenge in cancer management, being conventional chemotherapy the only therapeutic option^15^. It has been recently reported that TNBC cells with a mesenchymal phenotype secrete a greater amount of extracellular matrix proteins relative to non-mesenchymal cells and present basal levels of UPR activation^16^. Under these conditions, triggering the UPR may facilitate tumor cell survival and growth by increasing the expression of the ER chaperones, reducing the load of new synthesized proteins in the ER lumen, and by activating ER-associated degradation of unfolded proteins^17^. However, mesenchymal TNBC cells are markedly sensitive to apoptosis induced by different ER stress stimuli,^16^ although the mechanism underlying this cell death upon ER stress has not been elucidated.

In this work, we sought to determine the relative contribution of the extrinsic and intrinsic apoptotic pathways to the induction of cell death upon sustained ER stress in TNBC cells. Our results showed that both activating transcription factor-4 (ATF4)/TRAIL-R2/caspase-8 and Noxa-mediated pathways are involved in the cell death process induced by ER stress in TNBC cells. Our results also demonstrated that maintenance of cellular FLICE-inhibitory protein (FLIP) levels following ER stress plays an adaptive role to prevent early activation of the extrinsic apoptotic pathway in these tumor cells.

## Results

### ER stress induces cell death in TNBC cells through a mitochondria-operated apoptotic pathway

**Fig. 1:**
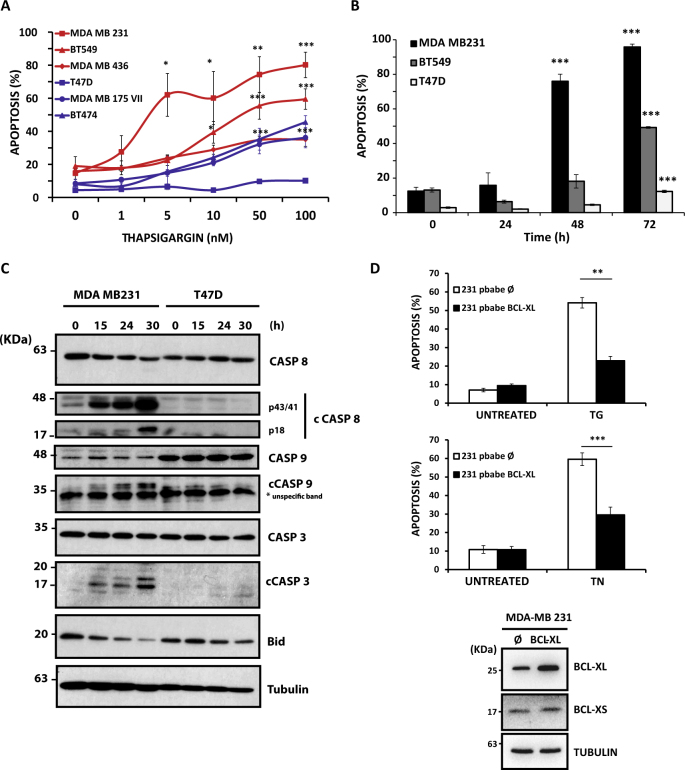
ER stress induces apoptosis specifically in TNBC cells through a mitochondria-operated pathway Apoptosis was measured after 3 days of treatment by subG1 analysis as described under Materials and methods section. Error bars represent standard error of the mean (SEM) from at least three independent experiments. *P < 0.05; **P < 0.01; ***P < 0.001. **A**) Dose–response of thapsigargin (TG) in apoptosis in TNBC (red lines) and non-TNBC (blue lines) cells. **B**) Kinetics of apoptosis induction by TG (100 nM) in TNBC (MDA-MB231 and BT549) and non-TNBC (T47D) cell lines. **C**) MDA-MB231 and T47D were treated with TG (100 nM) for the indicates times. Kinetics of caspase processing and Bid loss were determined in whole-cell extracts by western blotting. Tubulin levels were used as protein-loading controls. **D**) Apoptosis was assessed in Bcl-X_L_-overexpressing MDA-MB231 cells treated with 100 nM TG or 1 µg/ml tunicamycin (TN). Bcl-XL expression was assessed by western blotting. Tubulin expression was used as a protein-loading control

We first evaluated the sensitivity of different TNBC and non-TNBC cell lines to ER stress-induced apoptosis. Dose–response experiments with thapsigargin, a well-known ER stress inducer, show that TNBC cell lines of basal phenotype are more sensitive than luminal tumor cell lines to treatment with thapsigargin for 72 h (Fig. 1A) as previously reported^16^. We also determined the kinetics of apoptosis induced by thapsigargin in TNBC and non-TNBC cell lines. As shown in Fig. 1B, apoptosis was induced in TNBC cell lines MDA-MB231 and BT549 after 48 and 72 h treatment, respectively. In contrast, the non-TNBC cell line T47D was markedly resistant to thapsigargin-induced apoptosis. Cell death induced by thapsigargin was strongly inhibited in two TNBC cell lines, MDA-MB231 and BT549, when treated in the presence of the pan-caspase inhibitor Z-VAD-fmk (Fig. S1A). Likewise, cell death provoked by ER stress inducer tunicamycin was abrogated in the presence of the general caspase inhibitor Q-VD-OPh in both TNBC cell lines (Fig. S1B), further indicating the activation of caspase-dependent cell death upon ER stress in TNBC cells. Consistent with these findings, we also found the processing of various caspases and the loss of BH3-only protein Bid in MDA-MB231 cells treated with thapsigargin and not in cells of the non-TNBC cell line T47D (Fig. 1C).

To further characterize the mechanism underlying ER stress-induced apoptosis in TNBC cells, we assessed the role of the mitochondria-operated cell death pathway in apoptosis upon ER stress in MDA-MB231 cells ectopically expressing the antiapoptotic protein of the Bcl-2 family, Bcl-X_L_ (231-Bcl-X_L_). As shown in Fig. 1D, activation of apoptosis by ER stress inducers occurred through a mitochondria-operated pathway as it was substantially inhibited in 231-Bcl-X_L_ cells.

### Role of the intrinsic and extrinsic apoptotic pathways in cell death induced by ER stress in TNBC cells

**Fig. 2:**
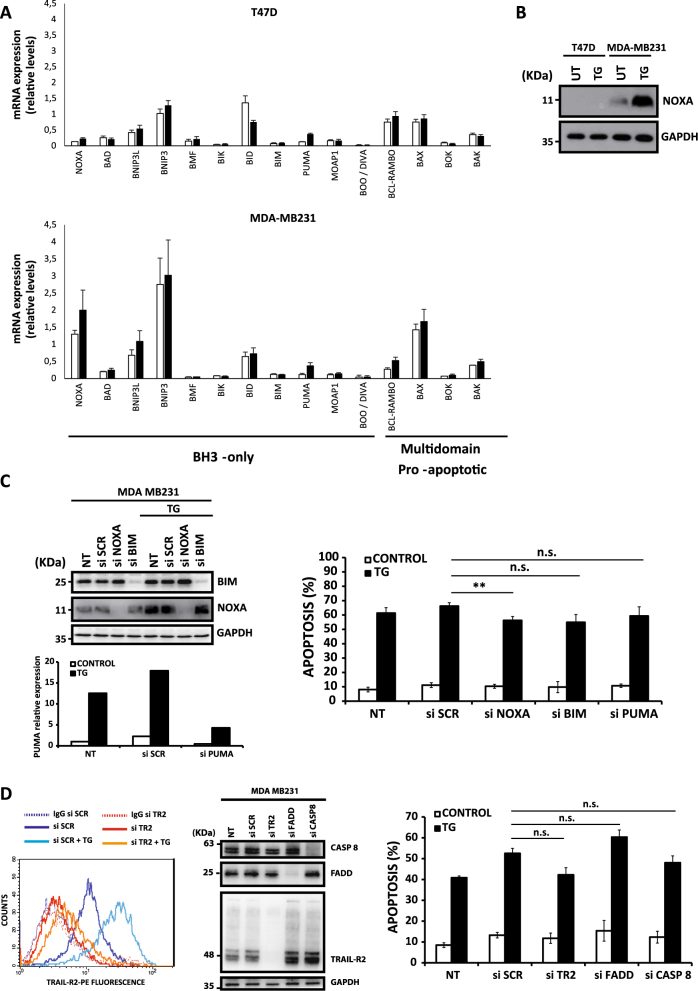
Role of the Bcl-2 protein family in cell death induced by ER stress **A**) MDA-MB231 and T47D breast cancer cells were treated with or without 100 nM thapsigargin (TG) for 20 h. The expression of Bcl-2 family genes was examined by RT-MLPA as described in materials and methods section. Error bars represent SD (*n* = 3) of the mRNA expression levels normalized by beta-2-microglobulin. **B**) Cells were treated with or without TG 100 nM for 24 h. Whole-cell extracts were prepared and Noxa expression was determined by western blotting. GAPDH expression was used as a protein-loading control. **C**) MDA-MB231 cells were either non-transfected (NT) or transfected with siRNAs for 30 h prior to treatment with 50 nM TG for 15 h. Noxa and Bim protein levels were assessed by western blotting. Puma mRNA levels was determined by RT-qPCR. Apoptosis was assessed in NT and transfected cells after 48 h of TG treatment (50 nM). Error bars represent SEM from at least three independent experiments. **P < 0.01; n.s. not statistically significant. **D**) MDA-MB231 cells were either NT or transfected with siRNAs for 30 h. TRAIL-R2 cell surface levels were determined by flow cytometry with TRAIL-R2-PE antibody as described in the material and methods section (left panel). Cells incubated with control IgG-PE antibody were used as a control for background fluorescence. Caspase-8, TRAIL-R2, and FADD levels were assessed by western blotting (middle panel). Data shown are representative of three independent experiments. In parallel, cultures of NT and siRNA-transfected cells were treated with 50 nM TG for 48 h, and apoptosis was determined by subG1 analysis (right panel). Error bars represent SEM from at least three independent experiments. n.s. not statistically significant

To assess the role of the Bcl-2 protein family in cell death regulation by ER stress in TNBC cells, we determined by RT-MLPA (retro transcription-multiplex ligand probe-dependent amplification) the expression levels of proapoptotic genes of the Bcl-2 family in MDA-MB231 and T47D cells treated with thapsigargin. Results of these experiments showed little variation in the messenger RNA (mRNA) expression levels of BH3-only and multi-domain proapoptotic proteins in response to thapsigargin (Fig. 2A). Interestingly, basal mRNA levels of proapoptotic genes such as Noxa, BNIP3, and BAX were clearly higher in ER stress-sensitive MDA-MB231 cells as compared to resistant T47D cells (Fig. 2A). As Noxa has been demonstrated to play a role in ER stress-induced apoptosis^18,19^, we determined the protein levels of Noxa in both MDA-MB231 and T47D cell lines in response to thapsigargin. As shown in Fig. 2B, TNBC MDA-MB231 cells express detectable Noxa protein levels, which were clearly upregulated following thapsigargin treatment. In contrast, in the non-TNBC breast tumor cell line T47D Noxa levels were undetectable, even after treatment with thapsigargin. To assess the role of BH3-only proteins in ER stress-induced apoptosis in TNBC cells, we silenced the expression of Noxa as well as other two BH3-only proteins that have been implicated in ER stress-induced cell death: Bim and Puma^9,18^. Despite a marked reduction in the target mRNA o protein levels (Fig. 2C, left panels), we only observed a slight inhibitory effect of Noxa small interfering RNA (siRNA) and no significant effect of Puma and Bim siRNAs on thapsigargin-induced apoptosis (Fig. 2C, right panel). Furthermore, simultaneous knockdown of Noxa, Bim, and Puma did not significantly reduce apoptosis upon thapsigargin treatment (Fig. S1C). Taken together, all these results suggest that the requirement of the mitochondrial apoptotic pathway in the activation of apoptosis by ER stress in TNBC cells does not depend exclusively on the expression of the BH3-only proteins Noxa, Bim, or Puma.

Protein kinase RNA-like ER kinase (PERK), a transmembrane kinase of the ER phosphorylates eukaryotic initiation factor 2α upon ER stress to attenuate general protein synthesis by inhibiting mRNA translation, thus reducing the protein burden in ER. However, translation of certain transcripts such as the mRNA for transcription factor ATF4 is favored under ER stress to activate an adaptive response^20^. After chronic ER stress, ATF4 is also involved in ER stress-induced apoptosis through the induction of transcription factor CAAT/enhancer binding protein homologous protein (CHOP)^21^. It has been reported that the proapoptotic function of the ATF4/CHOP axis involves the transcriptional regulation of various target genes including GADD34 phosphatase and ERO1alpha oxidase, which increases global protein synthesis and production of reactive oxygen species (ROS), respectively, leading to proteotoxicity in stressed cells^22^. However, treatment of MDA-MB231 cells with thapsigargin in the presence of the GADD34 phosphatase inhibitor salubrinal^23^ did not reduce the levels of apoptotic cells (Fig. S2A). Likewise, treatment of MDA-MB231 cells with thapsigargin in the presence of *N*-acetyl-cysteine, an antioxidant that decreases ROS and oxidative stress, did not inhibit apoptosis (Fig. S2B).

A potential CHOP-binding site has been identified in the promoter region of the proapoptotic TRAIL-R2/DR5 gene^12^, whose induction in response to ER stress has been observed in different tumor cell lines^11–13^. As shown in Fig. 2D (left panel), treatment of MDA-MB231 cells with thapsigargin markedly upregulated TRAIL-R2/DR5 expression at the cell surface. To examine the role of the TRAIL-R2-mediated signaling in ER stress-induced apoptosis in TNBC cells, we silenced the expression of the key proteins involved in the early signaling of TRAIL: TRAIL-R2/DR5, FADD, and caspase 8, prior to incubation with the ER stress inducer (Fig. 2D, left and middle panels). However, knockdown of any of these proteins of the extrinsic pathway of apoptosis did not block thapsigargin-induced apoptosis in MDA-MB231 cells (Fig. 2D, right panel). Likewise, knockdown of caspase-8 expression did not abrogate ER stress-induced apoptosis in MDA-MB231 cells treated either with dithiothreitol (DTT) or tunicamycin (Fig. S2C), two well-known inducers of ER stress. These results were further corroborated in the BT549 cell line, another TNBC cell line, in which silencing caspase-8 expression did not reduce ER stress-induced apoptosis (Fig. S2D).

### Simultaneous inhibition of the extrinsic and intrinsic apoptotic pathways prevents ER stress-induced apoptosis in TNBC cells

**Fig. 3:**
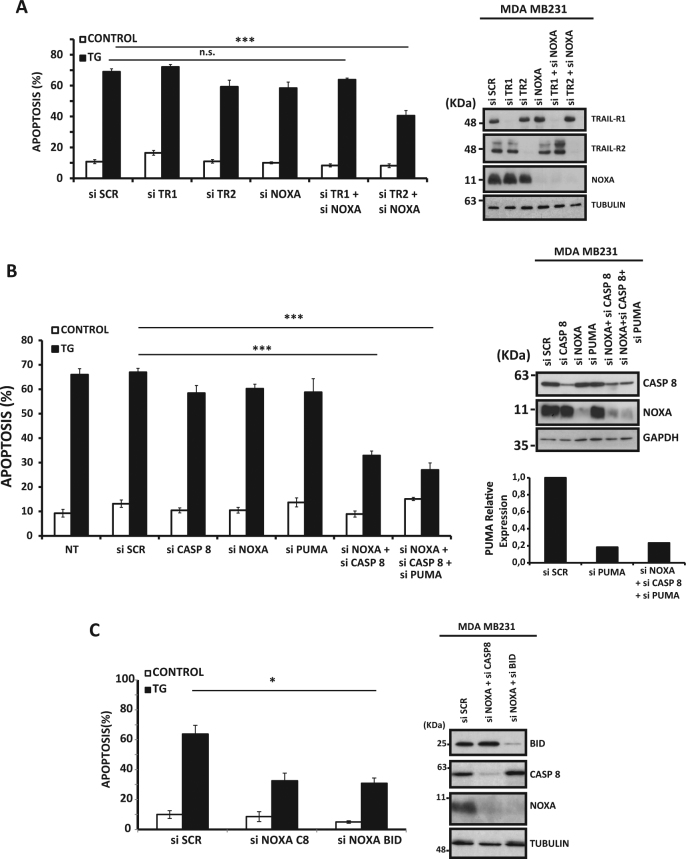
Simultaneous inhibition of the intrinsic and extrinsic apoptotic pathways blocks thapsigargin-induced apoptosis in TNBC cells MDA-MB231 cells were either non-transfected (NT) or transfected with the indicated siRNAs for 30 h to determine the role of **A**) TRAIL receptors (TRAIL-R1 and TRAIL-R2), **B**) caspase-8 (casp8) and **C**) Bid in apoptosis upon ER stress. Knockdown efficiency was determined by either western blotting or RT-PCR. Parallel cultures of NT or siRNA-transfected cells were then treated with 50 nM thapsigargin (TG) for 48 h, and apoptosis was then determined. Error bars represent SEM from at least three independent experiments. *P < 0.05; ***P < 0.001; n.s. not statistically significant

Given that, individually, none of the two major apoptotic pathways appeared to be involved in the regulation of cell death by ER stress, we determined the effect of inhibiting simultaneously both pathways on apoptosis induced by ER stress. Interestingly, combined silencing of Noxa and TRAIL-R2 expression, but not TRAIL-R1, significantly inhibited thapsigargin-induced apoptosis (Fig. 3A), further supporting a role of both intrinsic and extrinsic apoptotic pathways in cell death upon ER stress in MDA-MB231 cells. Importantly, though thapsigargin treatment induced the expression of TRAIL in MDA-MB231 cells (Fig. S3A), simultaneous silencing of TRAIL and Noxa did not prevent apoptosis upon ER stress (Fig. S3B), thus suggesting the activation of a ligand-independent apoptotic mechanism. Strikingly, simultaneous knockdown of Noxa and caspase-8 expression in MDA-MB231 cells substantially inhibited thapsigargin-induced apoptosis (Fig. 3B), which was not further reduced if PUMA expression was also silenced (Fig. 3B). In addition, combined silencing of Noxa and Bid, a BH3-only protein responsible for the amplification of death receptor signals by triggering the mitochondrial pathway of apoptosis^24^, resulted in a significant inhibition of thapsigargin-induced apoptosis (Fig. 3C). Further evidences for the involvement of both intrinsic and extrinsic pathways in thapsigargin-induced apoptosis were obtained from the analysis of caspase-8 processing in cells overexpressing Bcl-X_L_. In these cells, caspase-9 and caspase-3 activation upon thapsigargin treatment were markedly inhibited as compared to control pbabe cells (Fig. S3C). Importantly, although reduced, caspase-8 processing was still observed in thapsigargin-treated Bcl-X_L_ cells (Fig. S3C), these data suggesting the activation of caspase-8 upstream of mitochondria through death receptor activation. Supporting these data, similar results were obtained in cells treated with TRAIL (Fig. S3D). Thus apoptosis (upper panel) and caspase-8 processing (lower panel) induced by TRAIL were significantly reduced in Bcl-X_L_ cells (Fig. S3B). Together, these results indicate a dual activation of caspase-8 upon ER stress in MDA-MB231 cells, upstream of mitochondria following death receptor activation and downstream of mitochondria possibly through a caspase-9/caspase-3 axis. Collectively, these results reveal that, in TNBC cells, ER stress leads to the simultaneous activation of both intrinsic and ligand-independent, TRAIL-R2-dependent extrinsic apoptotic pathways, any of which is sufficient to kill the cells through a mitochondria-operated mechanism.

### Role of UPR sensors in ER stress-induced apoptosis in TNBC cells

**Fig.4:**
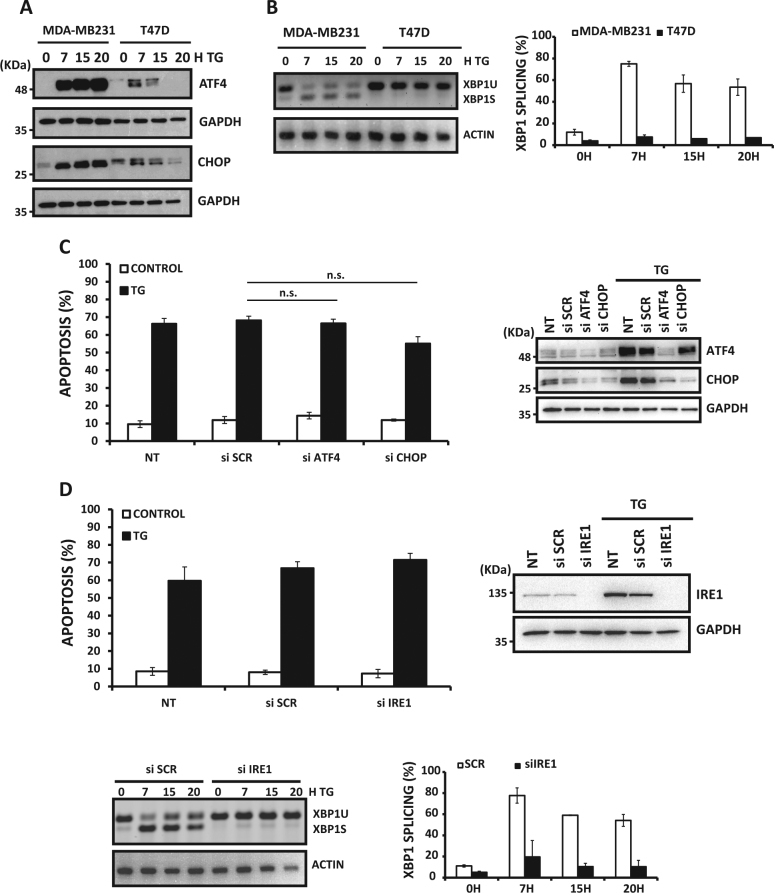
Role of UPR sensors in ER stress-induced apoptosis **a** MDA-MB231 and T47D breast cancer cells were treated with or without 100 nM thapsigargin (TG) for different times. ATF4 and CHOP protein expression was assessed by western blotting. **B**) Cells were treated as in **A**) and XBP1 splicing (XBP1s) was analyzed by RT-PCR and subsequently quantified with respect to XBP1 unspliced (XBP1u). Data shown are representative of three independent experiments. MDA-MB231 cells were either non-transfected (NT) or transfected with siRNAs against ATF4 or CHOP **C**) or a siRNA against Ire1 **D**) for 30 h and then treated with 50 nM TG either for 15 h (protein expression) or for 48 h (apoptosis determination). Error bars represent SEM from at least three independent experiments. n.s. not statistically significant. XBP1 splicing (XBP1s) was analyzed by RT-PCR and subsequently quantified with respect to XBP1 unspliced (XBP1u). Error bars represent ±SEM from at least three independent experiments

To further characterize the mechanism underlying the differential sensitivity of TNBC and non-TNBC cells to ER stress inducers, we examined the activation state of two branches of the UPR that are known to regulate cell fate under ER stress conditions^1^. Thus expression of ATF4 and its target gene CHOP were significantly upregulated by thapsigargin in MDA-MB231 cells as compared to what was observed in non-TNBC T47D cells (Fig. 4A). In addition, activation of the inositol-requiring protein-1 (IRE1) branch of the UPR, as determined by the processing of the mRNA encoding the transcription factor X box-binding protein 1 (XBP1u) (Fig. 4B), was markedly induced in MDA-MB231 cells. In contrast, processing of transcription factor XBP1u was not observed in T47D cells (Fig. 4B). These results confirmed a differential activation of the UPR between ER stress-sensitive and -resistant cell lines.

We next sought to determine the role of the UPR branches in apoptosis induced by ER stress-inducing agents in TNBC cells. We observed that transfection of MDA-MB231 cells with specific siRNAs for ATF4 and CHOP markedly inhibited ER stress-induced upregulation of these proteins (Fig. 4C, right panel): However, silencing ATF4 or CHOP expression did not reduce apoptosis induced by thapsigargin (Fig. 4C, left panel). As the proapoptotic role of IRE1 could be mediated in part by the activation of Jun N-terminal kinase (JNK) signaling^25^, we determined the effect of a JNK-specific inhibitor in apoptosis induced by ER stress in MDA-MB231 cells. Results shown in Fig. S4A indicate that inhibition of JNK activity did not prevent apoptosis induced upon thapsigargin treatment in MDA-MB231 cells. In addition, IRE1 knockdown (Fig. 4D, right panel) completely abrogated XBP1u processing (Fig. 4D, bottom panels) but did not inhibit apoptosis induced by thapsigargin (Fig. 4D, left panel). Furthermore, simultaneous inhibition of the PERK and IRE1 pathways had no effect on ER stress-induced apoptosis (Fig. S4B). Confirming these results, combined knockdown of IRE1 and proteins of the extrinsic (Caspase-8) or intrinsic (Noxa) apoptotic pathways did not inhibit thapsigargin-induced apoptosis in MDA-MB231 cells (Fig. S4C). Finally, silencing the expression of UPR sensor activating transcription factor-6 (ATF6) did not result in a reduction of apoptosis induced by thapsigargin (Fig. S4D).

**Fig. 5:**
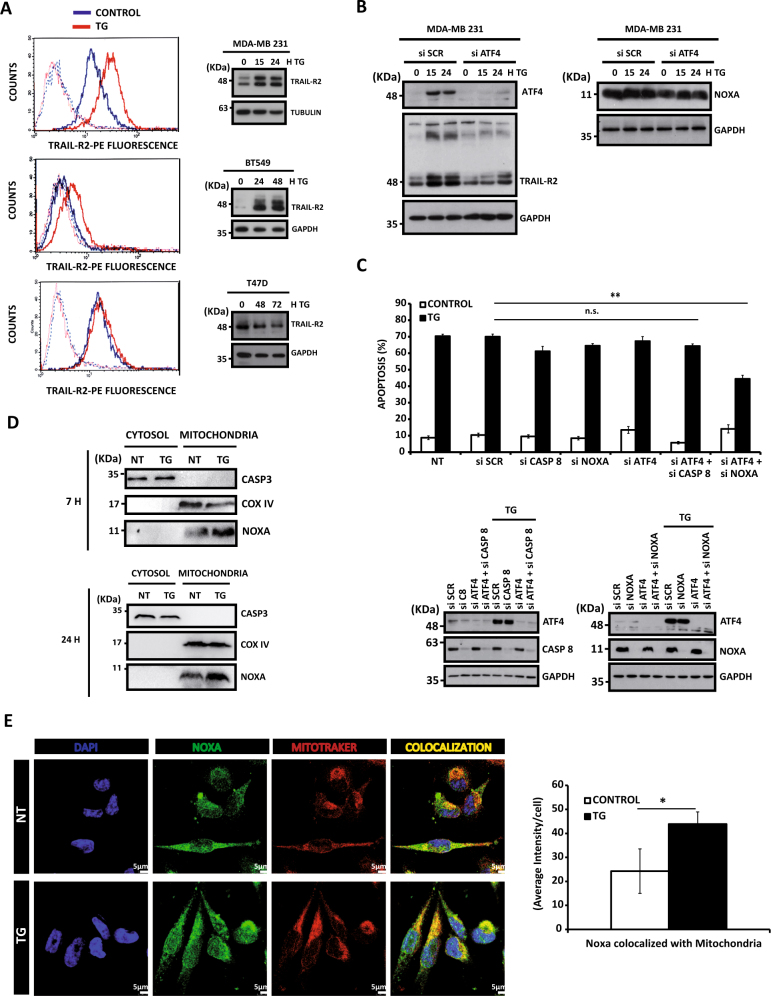
Involvement of the PERK branch of the UPR in the activation of the extrinsic apoptotic pathway upon ER stress **a** MDA-MB231, BT549 cells and T47D breast cancer cells were treated with or without thapsigargin (TG) 100 nM for 15 h and cell surface expression of TRAIL-R2 was determined by flow cytometry (left panel). Cells incubated with IgG-PE were used as a control for background fluorescence of cells. (Right panel) Cells were treated with 100 nM TG for the indicated times. TRAIL-R2 protein expression was assessed by western blotting. Data shown are representative of three independent experiments. **B**) MDA-MB231 cells were transfected with siRNA against ATF4 or a scrambled oligonucleotide (SCR) for 30 h and then treated with 50 nM TG for 15 or 24 h. ATF4, TRAIL-R2, and Noxa protein levels were assessed by western blotting. Data shown are representative of three independent experiments. **C**) MDA-MB231 cells were either non-transfected (NT) or transfected with siRNAs for 30 h and then treated with 50 nM TG for 48 h. Apoptosis was measured by subG1 analysis, as described in the materials and methods section. Error bars represent SEM from at least three independent experiments. **P < 0.01; n.s. not statistically significant. ATF4, Caspase-8, and Noxa levels were assessed by western blotting. **D**) MDA-MB231 cells were treated with or without (NT) 50 nM TG for 7 or 24 h. Noxa levels in cytosolic and mitochondrial fractions were assessed by western blotting as described under materials and methods section. Caspase-3 and COX IV were used as controls of cytosolic and mitochondrial protein, respectively **E**) MDA-MB231 cells were treated without (NT) or with 50 nM TG for 7 h and then processed for immunofluorescence analysis with Noxa antibody. Mitochondria were stained with MitoTracker. Representative confocal microscopic images are shown. Quantification of Noxa–mitochondria colocalization was performed with the Metamorph software. Error bars represent SEM from three independent experiments (100 cells/condition and experiment).*P < 0.05

Next, we determined the impact of inhibiting the PERK/ATF4/CHOP pathway on the expression of TRAIL-R2/DR5, a key effector in the activation of apoptosis upon ER stress in different cell systems^11–13^. Thapsigargin treatment elevated TRAIL-R2/DR5 protein expression (Fig. 5A, right panel) and cell surface levels (Fig. 5A, left panel) in both MDA-MB231 and BT549 cell lines but not in the ER stress-resistant T47D breast tumor cell line. Interestingly, ATF4 knockdown by siRNA prevented TRAIL-R2 upregulation upon thapsigargin treatment in MDA-MB231 cells (Fig. 5B). Although ATF4 may induce Noxa expression and thus triggers the intrinsic pathway of apoptosis in different systems^18,19,26^, ATF4 silencing did not significantly affect Noxa levels in thapsigargin-treated MDA-MB231cells (Fig. 5B). Collectively, these results indicate that, in the TNBC cells, the PERK pathway is responsible for the regulation of the extrinsic pathway of apoptosis upon ER stress while it does not seem to have a role in the pathway controlling Noxa levels.

The above data prompted us to investigate whether simultaneous activation of the PERK/ATF4 and the Noxa-regulated pathways may be responsible for the observed induction of apoptosis upon ER stress in TNBC cells. Although knockdown of either Noxa or ATF4 expression did not affect the sensitivity of MDA-MB231 cells to thapsigargin, combined silencing of both proteins significantly reduced apoptosis upon ER stress (Fig. 5C). On the contrary, simultaneous silencing of ATF4 and caspase-8 expression did not alter the sensitivity of MDA-MB231 cells to thapsigargin (Fig. 5C), further indicating that both proteins belongs to the same apoptotic pathway. To get further insight into the mechanism underlying Noxa-mediated activation of apoptosis in TNBC cells, we examined the subcellular localization of Noxa in cells treated with thapsigargin. Results shown in Fig. 5D, E demonstrate that in untreated cells Noxa localized mainly at the mitochondria as described in other cell types^27^. Strikingly, mitochondrial levels of Noxa further increased upon ER stress in TNBC cells as determined by both cell fractionation and confocal microscopy (Fig. 5D and 5E), and this was accompanied by Noxa-mediated downregulation of antiapoptotic Mcl-1 protein (Fig. S4E, S4F).

Overall, our data demonstrate that activation of the PERK/ATF4/CHOP/TRAIL-R2 pathway together with the enhanced Noxa levels at the mitochondria in response to thapsigargin would be responsible for the greater sensitivity of TNBC cells to ER stress-inducing agents.

### FLIP regulates cell death upon ER stress in TNBC cells

**Fig. 6:**
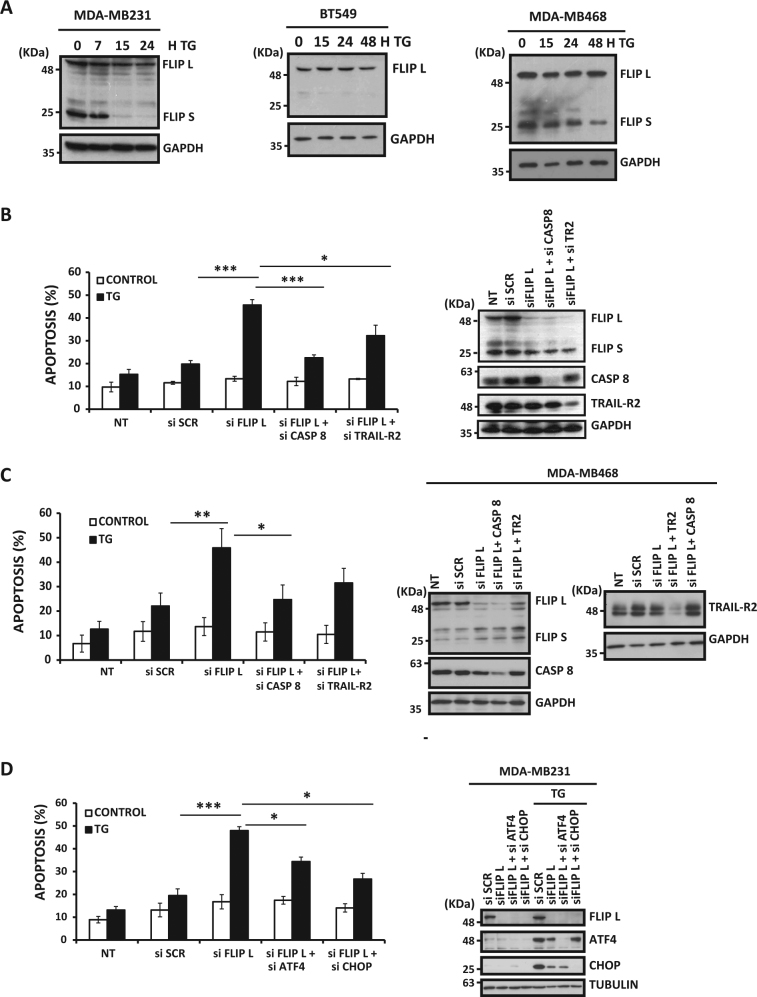
FLIP(L) controls ER stress-induced apoptosis **a** TNBC cells lines MDA-MB231, BT549, and MBA-MB468 were treated with 50 nM thapsigargin (TG) for the indicated times. FLIP protein levels were assessed by western blotting. **B**) MDA-MB231 TNBC cells were either non-transfected (NT) or transfected with siRNAs or SCR for 30 h and then treated with TG for 24 h. Apoptosis was measured by subG1 analysis. Error bars represent SEM from at least three independent experiments. *P < 0.05; ***P < 0.001. FLIP, Caspase-8, and TRAIL-R2 protein levels were assessed by western blotting. **C**) MDA-MB468 cells were transfected as in **B**), treated with TG for 48 h and then apoptosis was measured. Error bars represent SEM from at least three independent experiments. *P < 0.05; **P < 0.01. FLIP, Caspase-8, and TRAIL-R2 protein levels were assessed by western blotting. **D**) MDA-MB231 cells were either NT or transfected with siRNAs for 30 h and then treated with TG for 24 h. Apoptosis was measured by subG1 analysis. Error bars represent SEM from at least three independent experiments. *P < 0.05; ***P < 0.001. FLIP, ATF4, and CHOP protein levels were assessed by western blotting after 15 h of TG treatment

cFLIP proteins are key inhibitors of the extrinsic apoptotic pathway^28^. In addition, cFLIP downregulation has been observed in different cell types following ER stress^11,29^. To examine the role of cFLIP in the response of TNBC cells to ER stress, we first determined cFLIP levels in several TNBC cell lines treated with thapsigargin for different times (Fig. 6A). Although FLIP(S) levels declined upon ER stress, FLIP(L) expression and apoptosis remained unaffected (Fig. 6A) at times when TRAIL-R2 levels are already increased (Fig. 5A). To assess the potential adaptive role of FLIP(L) in TNBC cells undergoing ER stress, we determined the apoptotic response to thapsigargin in cells in which FLIP(L) was downregulated with siRNA prior to thapsigargin treatment. Remarkably, FLIP(L) silencing accelerated thapsigargin-induced apoptosis as compared to cells transfected with an irrelevant control oligonucleotide (Fig. 6B). Furthermore, in FLIP(L) knockdown cells, apoptosis induced by thapsigargin was significantly reduced if caspase-8 or TRAIL-R2/DR5 expression was also silenced (Fig. 6B), further demonstrating the involvement of the extrinsic apoptotic mechanism in this cell death process. These results were also confirmed in the TNBC MDA-MB468 cell line in which the reduction in FLIP levels by siRNA significantly increased the sensitivity of these cells to thapsigargin through activation of the extrinsic apoptotic pathway (Fig. 6C). In addition, concomitant silencing of FLIP(L) and proteins of the PERK branch of the UPR lead to abrogation of ER stress-induced apoptosis, further supporting the role of this UPR pathway in the activation of the extrinsic apoptotic program in TNBC cells (Fig. 6D). Overall, our data support that FLIP(L) might be playing a role in maintaining the adaptive response of TNBC cells during ER stress by preventing the early activation of apoptosis through proapoptotic TRAIL-R2, thus favoring cell survival and allowing adaptation to the stress-inducing conditions.

## Discussion

Pharmacological overactivation of the proapoptotic branches of the UPR in tumor cells that are prone to ER stress may represent a potentially relevant strategy to therapeutically target these cells^30^. TNBC cells with a basal-like and a mesenchymal phenotype show increased synthesis and secretion of extracellular matrix proteins, which results in an enhanced vulnerability to ER stress-inducing agents^16^, although the underlying cell death mechanism remains largely unknown. In this work, we have investigated the role of the intrinsic and extrinsic apoptotic pathways in the cell death process activated upon ER stress in TNBC cells. Our results demonstrate that in TNBC cells ER stress-inducing agents activates a mitochondria-operated apoptotic process through both the ATF4-dependent upregulation of proapoptotic TRAIL-R2/DR5 and the activation of a Noxa-mediated pathway.

Regarding the mechanism underlying the different sensitivity to ER stress observed between TNBC and luminal tumor cell lines, our data show marked differences in the activation of the PERK and IRE1 branches of the UPR. In this respect, our results indicate that differential activation of the PERK/ATF4/CHOP pathway may be, at least in part, responsible for the increased sensitivity of TNBC to ER stress-inducing agents. In contrast to previous data suggesting a certain role of the IRE1 branch in ER stress-induced apoptosis in different cell types^7,13,31^, our results do not support a role of IRE1 pathway in ER stress-induced apoptosis in TNBC cells. Our findings are consistent with prior work in breast epithelial cells expressing a constitutively active Her2/ErbB2 oncogene in which ER stress-induced apoptosis was independent of the IRE1 pathway^11^. Collectively, these data suggest that IRE1 function in the regulation of apoptosis after sustained ER stress may be cell type specific.

Transcription factors ATF4 and CHOP are important effectors in the activation of apoptosis following ER stress in different cell types through the regulation of the expression levels of various members of the Bcl-2 family. Thus activation of the ATF4/CHOP axis can either reduce Bcl-2 levels^32^ or increase the expression of some proapoptotic members of the Bcl-2 family, such as Bim, Puma, and Noxa^9,19,26,33^. Instead, our results show that in TNBC cells the PERK/ATF4/CHOP pathway is licensing apoptosis mainly through activation of the TRAIL-R2/caspase-8 pathway. These data are consistent with previous studies demonstrating that ER stress induces cell death by CHOP-mediated upregulation of TRAIL-R2 expression, leading to a ligand-independent, intracellular death-inducing signaling complex formation and caspase-8 activation^11–13^. In this respect, ectopic expression of TRAIL-R2 is sufficient to induce apoptosis in the absence of ligand^34^, most likely by the homotypic association of receptors through PLAD-like domains in TRAIL-R2^35^.

Regarding the activation of the intrinsic apoptotic pathway upon ER stress in TNBC cells, our observations clearly demonstrate a key role of BH3-only protein Noxa in mediating apoptosis in a redundant manner with TRAIL-R2/caspase-8 activation. TNBC cells show elevated basal levels of Noxa with a preferential localization in mitochondria that increases upon ER stress. Although transcriptional upregulation of Noxa expression has been proposed as a mechanism involved in apoptosis induced by ER stress in tumor cells^26^, posttranslational mechanisms may also be important^19,36^. Indeed, cytosolic retention of Noxa by phosphorylation has been reported to suppress its proapoptotic function^36,37^. Our results also demonstrate a Noxa-dependent downregulation of Mcl-1 in response to ER stress. Accordingly, Noxa has been shown to specifically interact with antiapoptotic Mcl-1 to induce its degradation by the proteasome^38^. Together, our data suggest that, under chronic ER stress in TNBC cells, upregulation of Noxa levels at the mitochondria will antagonize Mcl-1 function by promoting its proteasomal degradation, leading to mitochondria outer membrane permeabilization and the release of apoptogenic factors^39^.

Both FLIP(L) and FLIP(S) are short-lived inhibitory proteins^40^ expressed at high levels in breast cancers^41^. Evidences from different studies have indicated that regulation of FLIP expression levels is a key event in the mechanism controlling sensitivity of tumor cells to death receptor-mediated apoptosis. Thus selective suppression of FLIP expression in breast tumor cells induces caspase-8-dependent apoptosis both in vitro and in vivo^42^ and sensitizes these cells to TRAIL^43^. Our results reveal for the first time a key role of FLIP(L) in the preservation of cell viability under ER stress in TNBC cells and point at the deregulation of the mechanisms controlling FLIP levels^44^ as an essential event in the process leading to apoptosis under chronic ER stress^11^.

**Fig. 7:**
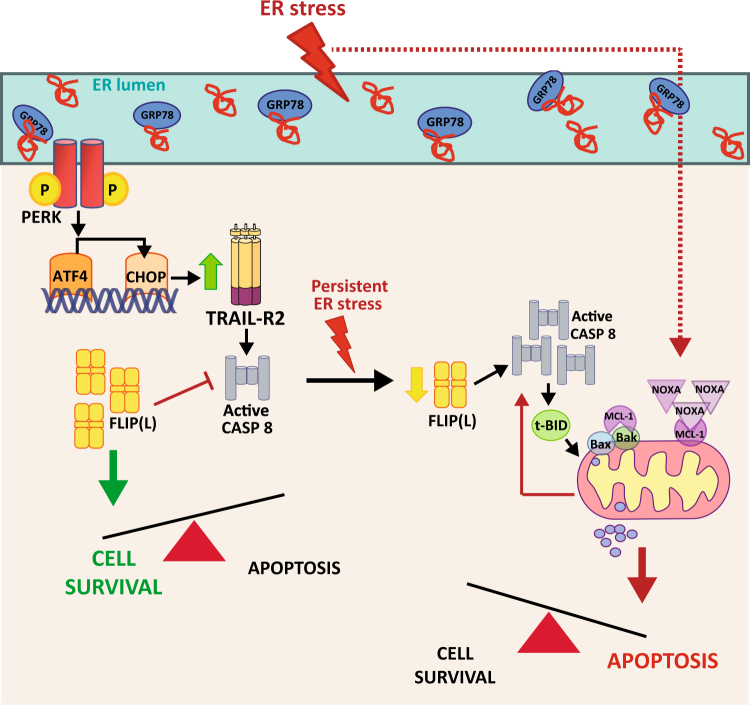
Schematic overview of the proposed mechanism of ER stress-induced apoptosis in TNBC cells ER stress induces cell death in TNBC cells through a FLIP(L)-regulated pleiotropic mechanism, which involves both caspase-8-dependent and -independent apoptotic pathways

To facilitate their migration, basal TNBC cells with a mesenchymal phenotype must secrete matrix proteases and scaffolding proteins to remodel the extracellular matrix. Secretory phenotype of mesenchymal TNBC cells relies on the basal activation of the PERK branch of the UPR as part of an adaptive response to preserve cell viability^16^. In this context, FLIP levels may play a key role in cell fate decisions as maintenance of FLIP levels will prevent early activation of death receptor-activated apoptotic pathway, thus providing time for the cell to adapt to the stressful conditions of the microenvironment (Fig. 7). However, if the stress persists, FLIP(L) levels may decrease leading to activation of the extrinsic apoptotic pathway, which, together with the activation of the Noxa-regulated intrinsic pathway, will execute the apoptosis program induced by the UPR.

## Materials and methods

### Cell culture

MDA-MB231 cells were maintained in RPMI medium supplemented with 10% fetal bovine serum, 2 mM L-glutamine, penicillin (50 U/ml) and streptomycin (50 μg/ml). T47D cells were grown in RPMI medium supplemented with 10% fetal bovine serum, 2 mM L-glutamine, 5 mM glucose, penicillin (50 U/ml), and streptomycin (50 μg/ml). MDA-MB468, MDA-MB175 VII, and BT549 cells were maintained in Dulbecco’s modified Eagle’s medium (DMEM) medium supplemented with 10% fetal bovine serum, 2 mM L-glutamine, penicillin (50 U/ml), and streptomycin (50 μg/ml). BT20 and MDA-MB436 cells were maintained in DMEM/F12 medium supplemented with 10% fetal bovine serum, 2 mM L-glutamine, penicillin (50 U/ml), and streptomycin (50 μg/ml). Cells were grown at 37 °C in a 5% CO_2_-humidified, 95% air incubator.

### Reagents and antibodies

Tunicamycin, thapsigargin, 4,6-diamidino-2-phenylindole (DAPI), and anti-α-tubulin were from Sigma-Aldrich (St. Louis, MO, USA). Anti-caspase 8 antibody was generously provided by Dr. Gerald Cohen (Leicester University, UK). Anti-FADD, anti-Bid, anti-caspase-3, and anti-IgG-PE antibodies were purchased from BD Bioscience (Erembodegem, Belgium). Anti-caspase 9 was purchased from MBL Life Science (Nagoya, Japan). Anti-TRAIL-R2-PE monoclonal antibody for surface receptor analysis was from Biolegend (San Diego, CA, USA). Anti-ATF4 and anti-GAPDH antibodies were from Santa Cruz Technology (Santa Cruz, CA, USA). Anti-CHOP, anti-TRAIL, anti-cleaved caspase-3, anti-cleaved caspase-8, anti-cleaved-caspase-9, anti-Bcl-XL, and anti-IRE1 antibodies were from Cell Signaling Technology (Temecula, CA, USA). Anti-TRAIL-R1 and anti-TRAIL-R2 antibodies were from R&D system (Minneapolis, USA). Anti-FLIP (NF6) antibody was from Adipogen (San Diego, CA, USA). Anti-Noxa and anti-Bim were from Calbiochem (Darmstadt, Germany) Anti-Noxa antibody for immunofluorescence was from Abcam (Cambrige, UK). Donkey anti-rabbit Cy3 and donkey anti-mouse Alexa488 secondary antibodies were from Jackson ImmunoResearch (West Grove, PA, USA). Horseradish peroxidase or fluorescein isothiocyanate-conjugated secondary antibodies, goat antimouse, and goat anti-rabbit antibodies were from DAKO (Cambridge, UK). DTT was from Roche (Basel, Switzerland). Q-VD was from Apexbio (Hsinchu, Taiwan). Z-VAD-fmk was from Bachem AG (Bubendorff, Switzerland). MitoTracker was purchased from Fisher Scientific (Waltham, MA, USA).

### Determination of apoptosis

Cells (3 × 10^5^/well) were treated in 6-well plates. After treatment, hypodiploid apoptotic cells were detected by flow cytometry according to published procedures^11^.

### Analysis of TRAIL receptors by flow cytometry

Cells were detached with trypsin solution and resuspended in growth media. After incubation for 15 min under cell culture conditions (37 °C in a 5% CO_2_-humidified, 95% air incubator), cells were washed with ice-cold phosphate-buffered saline (PBS) and resuspended in PBS. Cells were then labeled either with 5 μg/ml of anti-TRAIL-R2-PE or an IgG-PE control antibody for 30 min on ice and darkness. Quantitative analysis of the receptor cell surface expression was carried out in a FACSCalibur cytometer using the Cell Quest Software (Becton Dickinson, Mountain View, CA, USA).

### Immunofluorescence analysis

Cells were grown on coverslips, fixed in paraformaldehyde 4% at room temperature for 5 min and then permeabilized with 100% methanol at −20 °C for 5 min. Cells were incubated with primary antibodies at 4 °C overnight, washed with 0.1% PBS-Tween, and incubated with the appropriate fluorescent secondary antibody for 1 h. Nuclei were stained with DAPI (1 μg/ml) after secondary labeling.

### Confocal microscopy and image analysis

Confocal images were captured using TCS SP5 confocal Leica laser scanning systems equipped with DMI60000 microscope. Image processing was carried out using the Leica (LAS) and Adobe Photoshop software. For presentation, whole images were adjusted for intensity level, contrast, and/or brightness. Quantification of intracellular Noxa or mitochondria was performed using the Metamorph Offline software, measuring average intensity of Noxa over mitochondria.

### Immunoblot analysis of proteins

Cells (3 × 10^5^) were washed with PBS and lysed in TR3 lysis buffer (3% sodium dodecyl sulfate (SDS), 10% Glycerol, 10 mM Na_2_HPO_4_). Then lysates were sonicated and protein content was measured with the Bradford reagent (Bio-Rad Laboratories, USA), before adding Laemmli sample buffer. Proteins were resolved on SDS-polyacrylamide minigels and detected as described previously^11^. Tubulin and GAPDH (glyceraldehyde 3-phosphate dehydrogenase) were used as protein-loading controls.

### RNA interference

siRNAs against ATF4 (5ʹ-GCCUAGGUCUCUUAGAUGA-3ʹ), ATF6 (5ʹ-GCAACCAAUUAUCAGUUUA-3ʹ), Bim (5ʹ-GCAACCUUCUGAUGUAAGU-3ʹ), Casp8 (5ʹ-GGAGCUGCUCUUCCGAAUU-3ʹ), BID (5ʹ-AGACAAUGUUAAACUUAUA-3ʹ), CHOP pool (5ʹ-AGGGAGAACCAGGAAACGGAA-3ʹ, 5ʹ-ACGGCUCAAGCAGGAAAUCGA-3ʹ, 5ʹ-AAGGAAGUGUAUCUUCAUACA-3ʹ, 5ʹ-CAGCUUGUAUAUAGAGAUUGU-3ʹ), FADD (5ʹ-GAAGACCUGUGUGCAGCAU-3ʹ), FLIP(L) (5ʹ-CCUAGGAAUCUGCCUGAUA-3ʹ), Ire1 (5ʹ-GCGUCUUUUACUACGUAAU-3ʹ), Noxa (5ʹ-GGUGCACGUUUCAUCAAU3ʹ), Puma (5ʹ-GGAGGGUCCUGUACAAUCU-3ʹ), TRAIL-R1 (5ʹ-GGUAACUUUCCGGAAUGACA-3ʹ), TRAIL-R2 (5ʹ-GAUCCCUUGUGCUCGUUGUC-3ʹ), TRAIL#1 (5ʹ-GCAGCUCACAUAACUGGGA-3ʹ), TRAIL#2 (5ʹ-GAAUAUGGACUCUAUUCCA-3ʹ), and non-targeting scrambled oligonucleotide (5ʹ-GAGCGCUAGACAAUGAAG-3ʹ) were synthesized by Sigma (St. Louis, MO). Cells were transfected with siRNAs using DharmaFECT-1 (Dharmacon) as described by the manufacturer. After 6 h, transfection medium was replaced with regular medium and cells were further incubated for 24 h before further analysis.

### Real-time quantitative PCR (qPCR)

mRNA expression was analyzed in triplicate by reverse transcriptase-qPCR on the ABI Prism7500 sequence detection system using predesigned assay-on-demand primers and probes (Applied Biosystems). Hypoxanthine-guanine phosphoribosyltransferase (HPRT1 Hs01003267_m1) was used as an internal control and mRNA expression levels of ATF6, TRAIL, and Puma were given as fraction of mRNA levels in control cells. Primers and probes used were: ATF6 (Hs00232586_m1), TRAIL (Hs00921974_m1), and Puma (Hs0024075_m1).

### Retroviral vectors and virus production

pbabe-BCL-xL was a gift from Dr. Cristina Muñoz (IDIBELL, Barcelona, Spain). Retroviruses for protein overexpression were produced by transfection of HEK293-T cells by the calcium phosphate method with the corresponding retroviral vectors. Retrovirus-containing supernatants were collected 48 h after transfection and concentrated by ultracentrifugation at 22,000 rpm for 90 min at 4 °C.

### Retro transcription-multiplex ligand probe-dependent amplification

RNA was isolated from cultured 3–5 × 10^6^ cells by the RNeasy Micro Kit (Qiagen GmbH, Hilden, Germany) according to the manufacturer’s protocol. RNA was analyzed by RT-MLPA using SALSA MLPA KIT R011-C1 Apoptosis mRNA from MRC-Holland (Amsterdam, The Netherlands) for the simultaneous detection of 40 mRNA molecules, including apoptosis-related genes. In brief, RNA samples (200 ng total RNA) were first reverse transcribed using a gene-specific primer mix. The resulting cDNA was annealed overnight at 60 °C to the RT-MLPA probe mix. Annealed oligonucleotides were ligated by adding Ligase-65 (MRC-Holland) and incubated at 54 °C for 15 min. Ligation products were amplified by PCR (35 cycles, 30 s at 95 °C; 30 s at 60 °C, and 1 min at 72 °C) with one unlabeled and one FAM-labeled primer. The final PCR fragments amplified were separated by capillary electrophoresis on a 96-capillary ABI-Prism 3730XL Genetic Analyzer (Applied Biosystems/Hitachi, Carlsbad, CA, USA). Peak area and height were measured using the GeneMapper v3.0 analysis software (Applied Biosystems). Ratios of individual peaks relative to the sum of all peaks were calculated, resulting in the relative abundance of mRNAs of the genes of interest and normalized with respect to β2-microglobulin.

### Subcellular fractionation

After treatment, cells washed with PBS and lysed in 30 μl ice-cold lysis buffer (80 mM KCl, 250 mM sucrose, 500 μg/ml digitonin and protease inhibitors in PBS). Cell lysates were centrifuged for 5 min at 10,000 × *g* to separate the supernatant (cytosolic fraction) and pellet (mitochondria-containing fraction). Proteins from the supernatant and pellet were resolved on SDS-15% polyacrylamide gel electrophoresis, and Noxa, COX IV, and Casp3 was determined by western blotting analysis.

### Quantification of Mcl-1 and XBP1

Mcl1 and XBP1 were quantified with the Image Quant 5.2. software. Results were normalized to the loading control and referred to the untreated condition.

### Statistical analysis

All data are presented as the mean ± SEM of at least three independent experiments. The differences among different groups were determined by Student’s *t*-test. *P* < 0.05 was considered significant.

## Electronic supplementary material

Supplementary data
