# Supplementary material for: Involvement of both caspase-8 and Noxa-activated pathways in endoplasmic reticulum stress-induced apoptosis in triple-negative breast tumor cells
Source: Cell Death Dis. 2018 Jan 26;9(2):134. doi: 10.1038/s41419-017-0164-7 (PMC5833688; doi:10.1038/s41419-017-0164-7)
Supplement: Supplementary file 1 — Supplementary data [file 41419_2017_164_MOESM1_ESM.pdf]

Supplementary figure legends

Figure S1. Apoptosis was measured after 3 days-treatment by subG1 analysis as described under materials and methods. Error bars represent standard error of the mean (SEM) from at least three independent experiments. ***, P < 0.001. **(A)** Analysis of apoptosis in MDA-MB231 (left panel) and BT549 (right panel) cell lines treated with or without 50 nM or 100 nM thapsigargin (TG), respectively, in the presence or absence of z-VAD-fmk (50µM). **(B)** MDA-MB231 (left panel) and BT549 (right panel) cells were treated with or without tunicamycin (TN) (1µg/ml) in the presence or absence of Q-VD-OPh (20µM) before determination of apoptosis. **(C)** MDA-MB231 cells were transfected with the indicated siRNAs for 30 hours prior to treatment with 50 nM thapsigargin (TG) for 48 hours. Noxa and Bim protein levels were assessed by western-blotting. Puma mRNA levels was determined by RT-qPCR. Apoptosis was assessed in transfected cells by subG1 analysis.

Figure S2. (A) Analysis of apoptosis in MDA-MB231 cells treated for 48 hours with or without 50nM thapsigargin (TG) in the presence or absence of salubrinal (50µM) for 48 hours. Results depicted show the average and range from two independent experiments. Phospho-eIF2α and eIF2α protein levels were assessed by western blotting after 15 hours of thapsigargin and salubrinal treatment. **(B)** Cells were treated with or without thapsigargin (TG) and N-acetylcysteine (NAC) (10mM) for 48 hours. Apoptosis was measured by subG1 analysis. Results show the average and range from two independent experiments. **(C)** MDA-MB231 cells were either non-transfected (NT) or transfected with siRNA of Caspase-8 (CASP8) or Scrambled oligonucleotide (SCR) for 30 hours and then treated either with 5 mM dithiothreitol (DTT) (left

panel) or 500 ng/ml tunicamycin (TN) (right panel) for 48 hours to determine apoptosis. **(D)** BT549 cells were either non-transfected (NT) or transfected with siRNA of Caspase-8 (CASP 8) or a Scrambled oligonucleotide (SCR) as in (C) and then treated with 100 nM thapsigargin (TG) for 72 hours. Apoptosis was then measured by subG1 analysis. Error bars represent SEM from three independent experiments. n.s. (not statistically significant). Caspase-8 protein levels were assessed by Western blotting.

Figure S3. (A) MDA-MB231 cells treated with thapsigargin (50nM) for 24 hours. TRAIL mRNA levels were determined by RT-qPCR. Results depicted show the average and range from two independent experiments. **(B)** MDA-MB231 cells were transfected with either a scrambled oligonucleotide (SCR) or the indicated siRNAs for 30 hours and then treated with 50nM thapsigargin (TG) for 48 hours. Apoptosis was measured by subG1 analysis as described under materials and methods. Error bars represent standard error of the mean (SEM) from at least three independent experiments. n.s. (not statistically significant) **, $P < 0.01$. TRAIL protein levels were assessed by western blotting after 15 hours of thapsigargin treatment. Caspase-8 (CASP 8), and Noxa levels were determined in whole cell extracts by western-blotting. Tubulin expression was used as protein loading control. **(C)** Control and Bcl-x_L-overexpressing MDA-MB231 cells were treated with 50 nM thapsigargin (TG) for the indicated times. Kinetics of caspase expression and processing were assessed in whole cell extracts by western blotting. Bcl-x_L expression was examined by western-blotting. Tubulin expression was used as protein loading control. **(D)** Control and Bcl-XL-overexpressing MDA-MB231 cells were treated with TRAIL (500ng/ml). Apoptosis was measured by subG1 analysis. Results depicted show the

average and range from two independent experiments. Caspase-8 levels and processing were analyzed in whole cell extracts by western-blotting. Tubulin expression was used as a protein loading control.

Figure S4. (A) MDA-MB231 cells were treated with or without thapsigargin (TG) and JNK inhibitor SP600125 (25µM) for 48 hours. Apoptosis was then measured by subG1 analysis. Results depicted show the average and range from two independent experiments. Phospho-JNK and JNK protein levels were assessed by western blotting after 24 hours of SP600125 treatment. **(B)** MDA-MB231 cells were either non-transfected (NT) or transfected with siRNAs for 30 hours, treated with thapsigargin (TG) for 48 hours and apoptosis was then measured. Error bars represent SEM from at least three independent experiments. n.s. (not statistically significant). ATF4 and IRE-1 protein levels were assessed by Western blotting. **(C)** MDA-MB231 cells were either non-transfected (NT) or transfected with siRNAs for 30 hours and then treated with thapsigargin (TG) for 48 hours before measuring apoptosis. Results depicted show the average and range from two independent experiments. Ire1, Caspase-8, ATF4 and Noxa protein levels were assessed by Western blotting. **(D)** MDA-MB231 cells were either non-transfected (NT) or transfected with siRNA against ATF6 or scrambled oligonucleotide (SCR) for 30 hours, treated with thapsigargin (TG) for 48 hours and apoptosis measured. Results depicted show the average and range from two independent experiments. ATF6 mRNA levels was determined by RT-qPCR**.** **(E)** MDA-MB231 cells were treated with thapsigargin (TG) for 7, 15 and 24 hours. Mcl-1 expression was assessed by Western blotting. Mcl-1 protein levels were quantified with respect to Tubulin. Error bars represent SEM from three independent experiments. **(F)** MDA-

MB231 cells were transfected with siRNA against NOXA or a Scrambled oligonucleotide (SCR) for 30 hours and then treated with thapsigargin (TG) for 24 hours. Mcl-1 and Noxa expression were assessed by Western blotting. Mcl-1 protein levels were quantified with respect to GAPDH. Error bars represent SEM from three independent experiments.

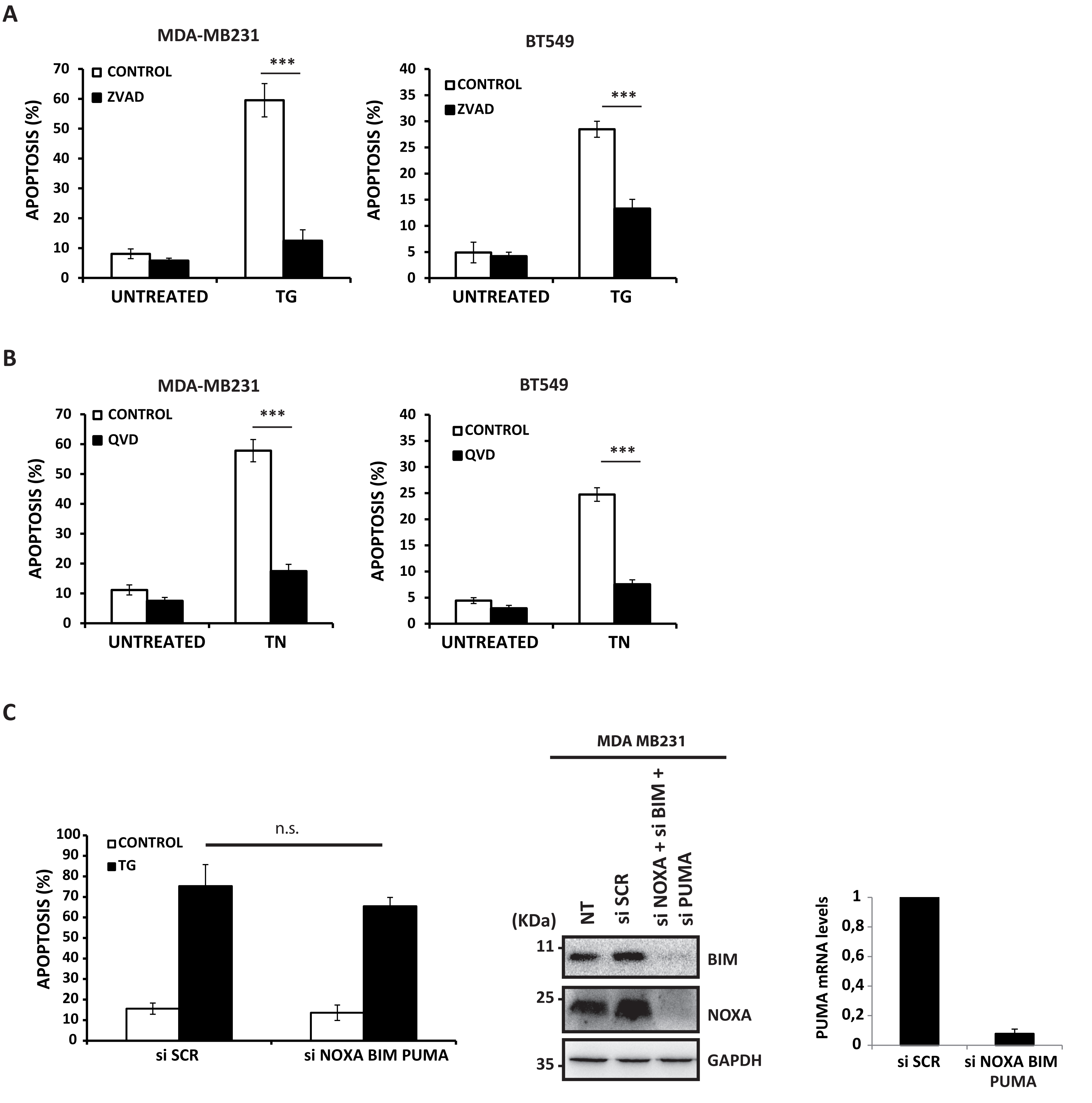
A
MDA-MB231
BT549
CONTROL
ZVAD
APOPTOSIS (%)
UNTREATED
TG
***
B
MDA-MB231
BT549
CONTROL
QVD
APOPTOSIS (%)
UNTREATED
TN
***
C
CONTROL
TG
n.s.
APOPTOSIS (%)
si SCR
si NOXA BIM PUMA
MDA MB231
(KDa)
NT
si SCR
si NOXA + si BIM + si PUMA
11
25
35
BIM
NOXA
GAPDH
PUMA mRNA levels
si SCR
si NOXA BIM PUMA

- FIGURE S1 -

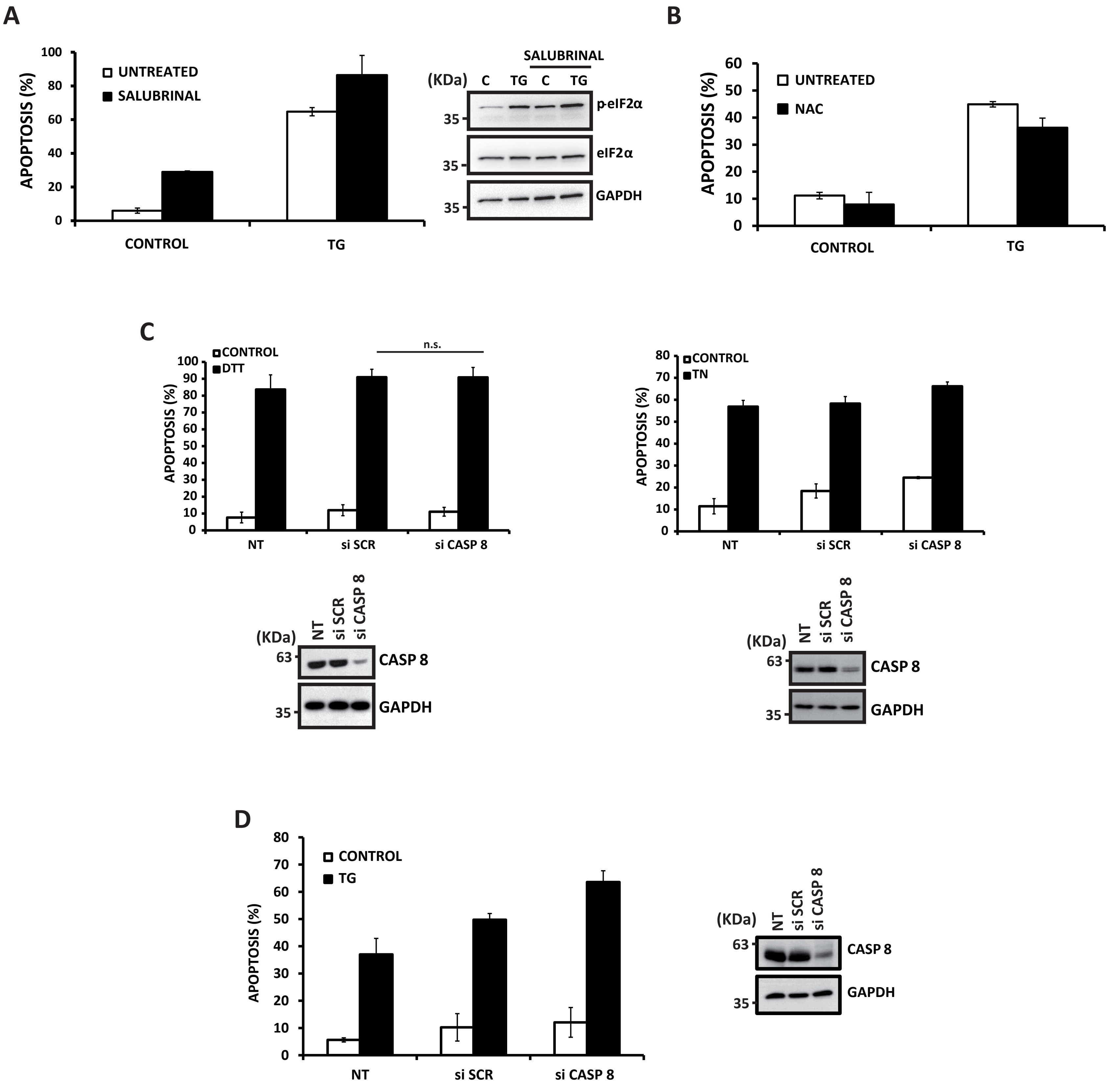
A
APOPTOSIS (%)
UNTREATED
SALUBRINAL
CONTROL
TG
SALUBRINAL
(KDa)
C
TG
C
TG
p-eIF2α
eIF2α
GAPDH
B
APOPTOSIS (%)
UNTREATED
NAC
CONTROL
TG
C
CONTROL
DTT
n.s.
APOPTOSIS (%)
NT
si SCR
si CASP 8
CONTROL
TN
CASP 8
GAPDH
D
CONTROL
TG
APOPTOSIS (%)
NT
si SCR
si CASP 8
(KDa)
CASP 8
GAPDH

- FIGURE S2 -

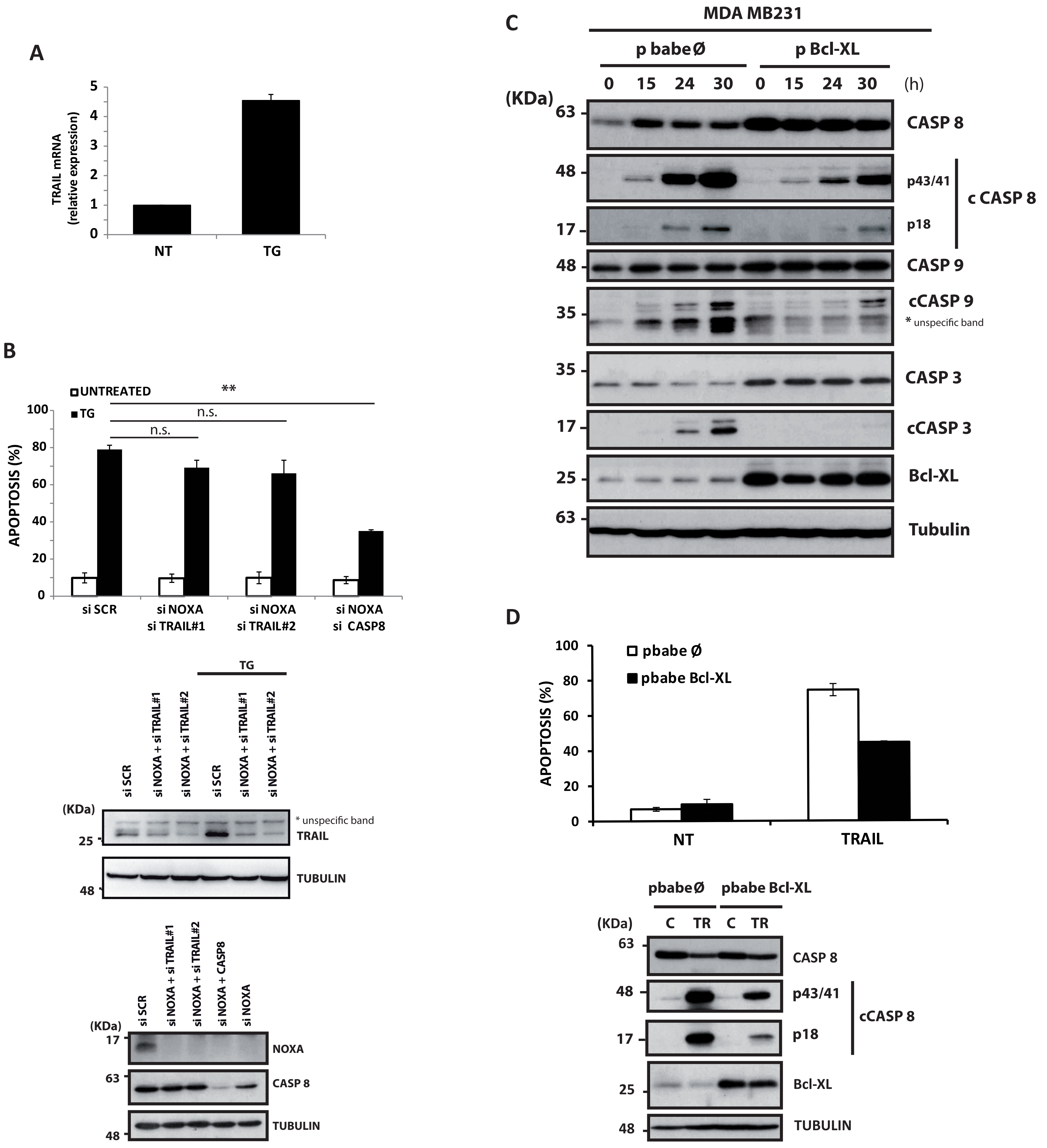
A
TRAIL mRNA (relative expression)
0
1
2
3
4
5
NT
TG
B
UNTREATED
TG
**
n.s.
n.s.
APOPTOSIS (%)
0
20
40
60
80
100
si SCR
si NOXA si TRAIL#1
si NOXA si TRAIL#2
si NOXA si CASP8
TG
si SCR
si NOXA + si TRAIL#1
si NOXA + si TRAIL#2
si SCR
si NOXA + si TRAIL#1
si NOXA + si TRAIL#2
(KDa)
25
48
* unspecific band
TRAIL
TUBULIN
si SCR
si NOXA + si TRAIL#1
si NOXA + si TRAIL#2
si NOXA + CASP8
si NOXA
(KDa)
17
63
48
NOXA
CASP 8
TUBULIN
C
MDA MB231
p babe Ø
p Bcl-XL
0
15
24
30
0
15
24
30
(h)
(KDa)
63
48
17
48
35
35
17
25
63
CASP 8
p43/41
p18
c CASP 8
CASP 9
cCASP 9
* unspecific band
CASP 3
cCASP 3
Bcl-XL
Tubulin
D
pbabe Ø
pbabe Bcl-XL
APOPTOSIS (%)
0
20
40
60
80
100
NT
TRAIL
pbabe Ø
pbabe Bcl-XL
(KDa)
C
TR
C
TR
63
48
17
25
48
CASP 8
p43/41
p18
cCASP 8
Bcl-XL
TUBULIN

- FIGURE S3-

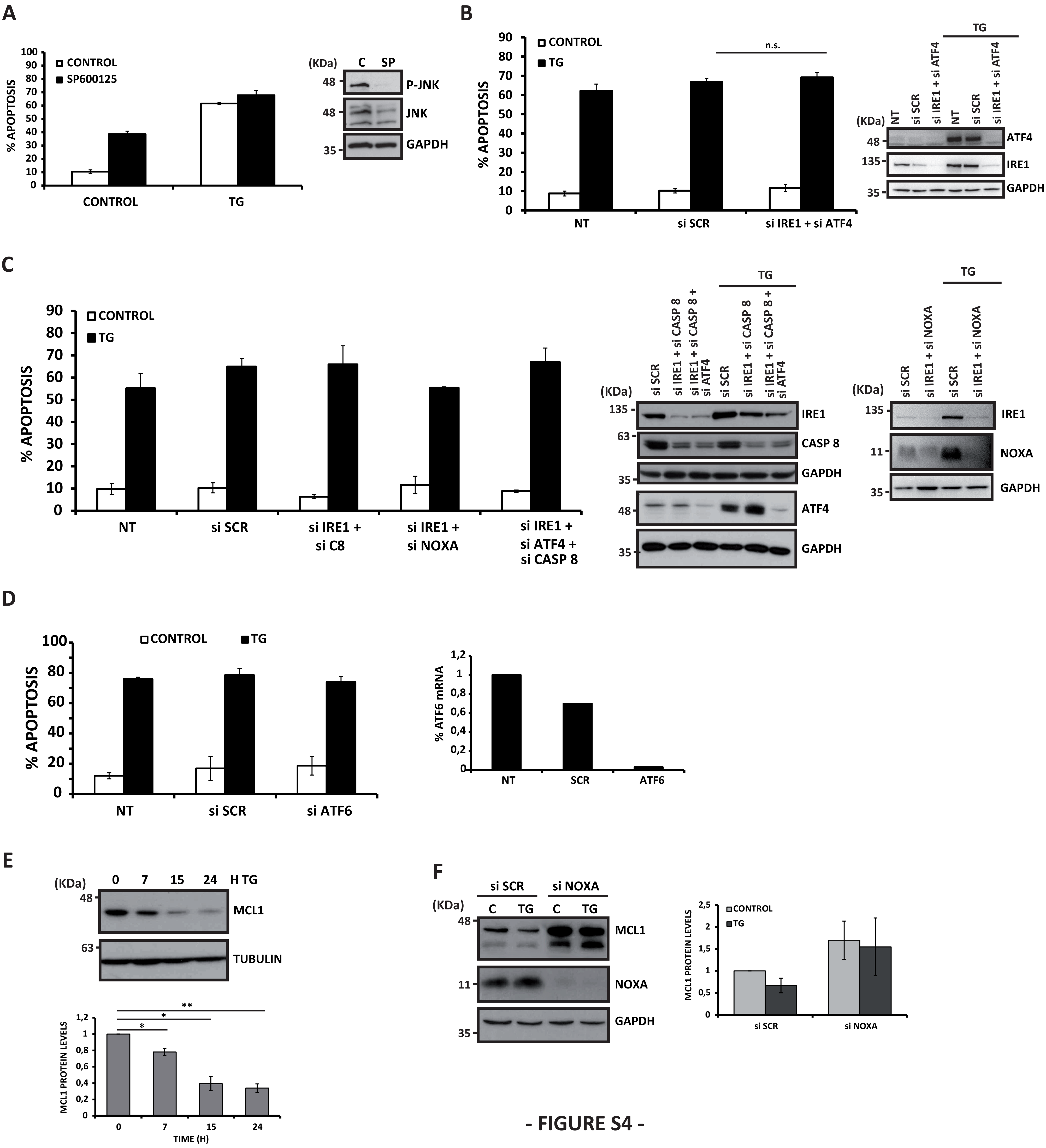

A
% APOPTOSIS
CONTROL
SP600125
CONTROL
TG
(KDa)
C
SP
48
P-JNK
48
JNK
35
GAPDH
B
% APOPTOSIS
CONTROL
TG
n.s.
NT
si SCR
si IRE1 + si ATF4
TG
(KDa)
NT
si SCR
si IRE1 + si ATF4
48
ATF4
135
IRE1
35
GAPDH
C
% APOPTOSIS
CONTROL
TG
NT
si SCR
si IRE1 + si C8
si IRE1 + si NOXA
si IRE1 + si ATF4 + si CASP 8
TG
(KDa)
si SCR
si IRE1 + si CASP 8
si IRE1 + si CASP 8 + si ATF4
135
IRE1
63
CASP 8
35
GAPDH
48
ATF4
35
GAPDH
TG
(KDa)
si SCR
si IRE1 + si NOXA
135
IRE1
11
NOXA
35
GAPDH
D
% APOPTOSIS
CONTROL
TG
NT
si SCR
si ATF6
% ATF6 mRNA
NT
SCR
ATF6
E
(KDa)
0
7
15
24
H TG
48
MCL1
63
TUBULIN
MCL1 PROTEIN LEVELS
TIME (H)
F
si SCR
si NOXA
(KDa)
C
TG
C
TG
48
MCL1
11
NOXA
35
GAPDH
CONTROL
TG
MCL1 PROTEIN LEVELS
si SCR
si NOXA

- FIGURE S4 -
